# Coagulofibrinolytic Changes in Patients with Post-cardiac Arrest Syndrome

**DOI:** 10.3389/fmed.2017.00156

**Published:** 2017-09-29

**Authors:** Takeshi Wada

**Affiliations:** ^1^Division of Acute and Critical Care Medicine, Department of Anesthesiology and Critical Care Medicine, Hokkaido University Graduate School of Medicine, Sapporo, Japan

**Keywords:** post-cardiac arrest syndrome, systemic ischemia/reperfusion, disseminated intravascular coagulation, no-reflow phenomenon, activation of coagulation, impaired anticoagulant, fibrinolytic shutdown

## Abstract

Whole-body ischemia and reperfusion due to cardiac arrest and subsequent return of spontaneous circulation constitute post-cardiac arrest syndrome (PCAS), which consists of four syndromes including systemic ischemia/reperfusion responses and post-cardiac arrest brain injury. The major pathophysiologies underlying systemic ischemia/reperfusion responses are systemic inflammatory response syndrome and increased coagulation, leading to disseminated intravascular coagulation (DIC), which clinically manifests as obstruction of microcirculation and multiple organ dysfunction. In particular, thrombotic occlusion in the brain due to DIC, referred to as the “no-reflow phenomenon,” may be deeply involved in post-cardiac arrest brain injury, which is the leading cause of mortality in patients with PCAS. Coagulofibrinolytic changes in patients with PCAS are characterized by tissue factor-dependent coagulation, which is accelerated by impaired anticoagulant mechanisms, including antithrombin, protein C, thrombomodulin, and tissue factor pathway inhibitor. Damage-associated molecular patterns (DAMPs) accelerate not only tissue factor-dependent coagulation but also the factor XII- and factor XI-dependent activation of coagulation. Inflammatory cytokines are also involved in these changes *via* the expression of tissue factor on endothelial cells and monocytes, the inhibition of anticoagulant systems, and the release of neutrophil elastase from neutrophils activated by inflammatory cytokines. Hyperfibrinolysis in the early phase of PCAS is followed by inadequate endogenous fibrinolysis and fibrinolytic shutdown by plasminogen activator inhibitor-1. Moreover, cell-free DNA, which is also a DAMP, plays a pivotal role in the inhibition of fibrinolysis. DIC diagnosis criteria or fibrinolysis markers, including d-dimer and fibrin/fibrinogen degradation products, which are commonly tested in patients and easily accessible, can be used to predict the mortality or neurological outcome of PCAS patients with high accuracy. A number of studies have explored therapy for this unique pathophysiology since the first report on “no-reflow phenomenon” was published roughly 50 years ago. However, the optimum therapeutic strategy focusing on the coagulofibrinolytic changes in cardiac arrest or PCAS patients has not yet been established. The elucidation of more precise pathomechanisms of coagulofibrinolytic changes in PCAS may aid in the development of novel therapeutic targets, leading to an improvement in the outcomes of PCAS patients.

## Introduction

The history of post-cardiac arrest syndrome (PCAS) dates back to the early 1970s. Negovsky named the unique and complicated pathophysiology of successfully resuscitated cardiac arrest, “post-resuscitation disease” (1). Since then, a bunch of investigations on this disease have been conducted, with concurrent progress in the management of cardiac arrest, including modern cardiopulmonary resuscitation (CPR) and emergency cardiovascular treatment. However, the long-term survival of resuscitated patients has not been associated with an improvement in the rate of return of spontaneous circulation (ROSC).

In 2008, the International Liaison Committee on Resuscitation suggested a new concept and definition of PCAS to indicate the direction of future research by evaluating and organizing the epidemiology, pathophysiology, therapy, and outcome of this disease (2). PCAS consists of four syndromes, which include systemic ischemia/reperfusion response and post-cardiac arrest brain injury. Coagulofibrinolytic changes are referred to as “increased coagulation,” which constitutes one “pathophysiology” of systemic ischemia/reperfusion response. The concept of “increased coagulation” is based mainly on the publications by Adrie et al., who suggested that the pathology of post-resuscitation was a “sepsis-like syndrome,” based on the evidence that successfully resuscitated patients showed several similar features, including a coagulation abnormality associated with systemic inflammatory response syndrome (SIRS), which is commonly seen in sepsis (3).

Around the same time as when post-resuscitation disease was suggested, the first study on disseminated intravascular coagulation (DIC) following cardiac arrest was reported (4). DIC is defined as the tissue factor-dependent systemic hypercoagulation, deficient control of coagulation by impaired physiologic anticoagulation systems, and plasminogen activator inhibitor-1 (PAI-1)-associated inhibition of fibrinolysis. These changes result in the thrombotic vascular occlusion, followed by an aggravation of the blood and oxygen supply to cells and tissues, ultimately leading to microvasculature damage and multiple organ dysfunction syndrome (MODS) (5–7). Interestingly, several years before the suggestion of post-resuscitation disease, the no-reflow phenomenon, characterized by impaired reperfusion after cerebral ischemia despite a stable systemic circulatory condition, was first reported by Ames et al (8). The severity of no-reflow depends on the duration of ischemia and is promoted by accompanying pathologies, including an activated coagulation system (8–12).

These findings imply that coagulofibrinolytic changes in patients with PCAS are deeply involved in post-cardiac arrest brain injury, which is the leading cause of death in PCAS patients. Thus far, however, there has been little evidence regarding the optimum therapeutic strategy for patients with coagulofibrinolytic changes associated with PCAS.

In this review, the pathophysiology of coagulofibrinolytic changes associated with PCAS is summarized based on the previous findings. The prediction of the outcomes of PCAS patients and therapeutic strategies targeting coagulofibrinolytic disorders associated with PCAS are also reviewed.

## Pathophysiology

Whole-body ischemia/reperfusion due to cardiac arrest and subsequent ROSC can produce SIRS, which is characterized by the release of systemic pro-inflammatory cytokines and generalized activation of leukocytes and endothelial cells. Recent advances in immunology have elucidated the involvement of innate immunity for the process of SIRS (13). Damage-associated molecular patterns (DAMPs), which are molecules derived from stressed or damaged cells and tissues, are sensed by pattern-recognition receptors expressed on immune cells and endothelial cells. These sensed signals lead to the production of pro-inflammatory cytokines, which stimulates the production of inflammatory biomarkers (14).

Close interaction among coagulation, inflammation, and innate immunity have been well investigated. Under normal conditions, the endothelium provides an anticoagulated surface including antithrombin, protein C/thrombomodulin system, and tissue factor pathway inhibitor (TFPI), along with the endothelial release of tissue-type plasminogen activator (t-PA), which dissolves forming clots. Coagulation and fibrinolysis after cardiac arrest are not adequately balanced due to the release of these inflammatory cytokines and DAMPs (12, 15).

**Figure 1:**
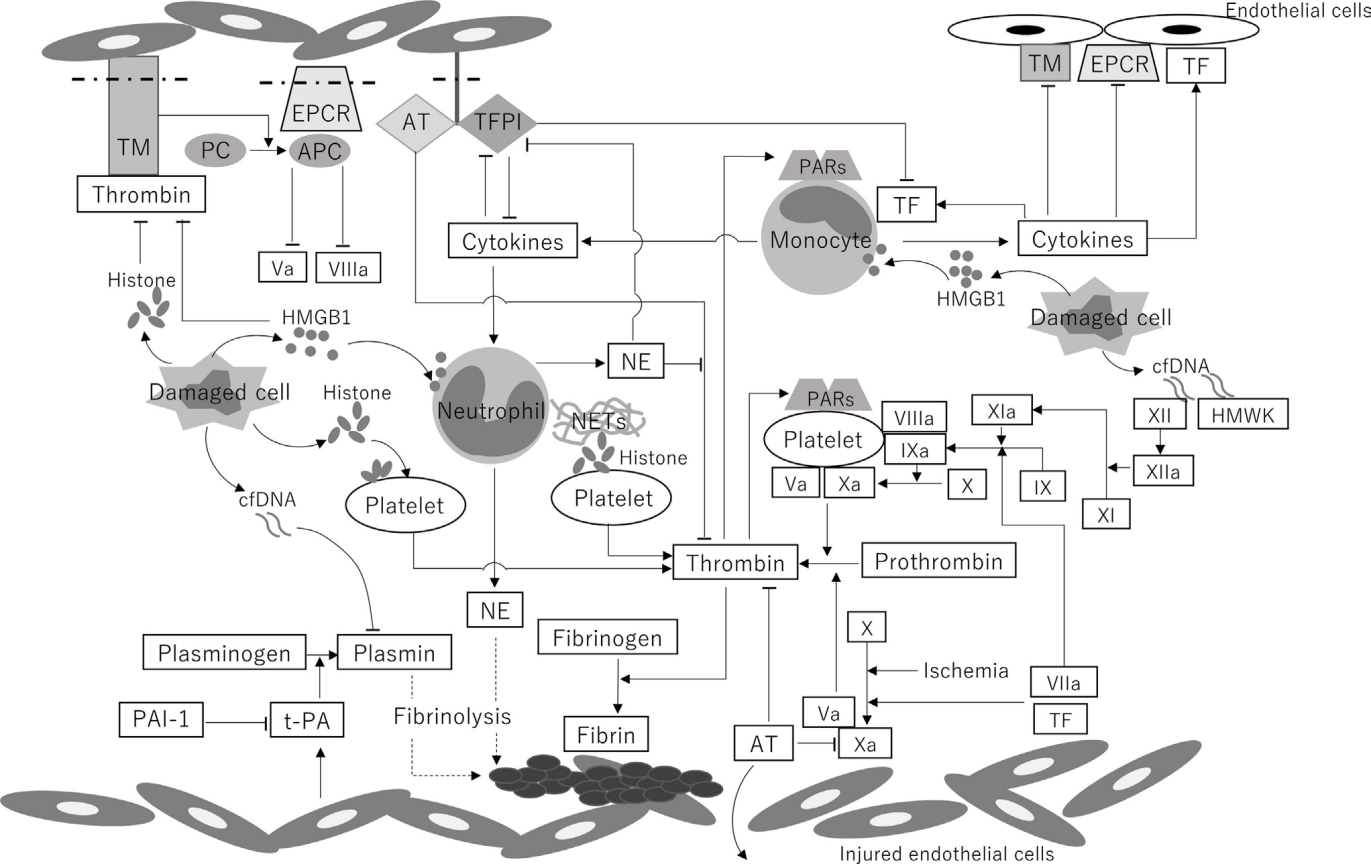
A schematic illustration of the coagulofibrinolytic changes in patients with PCAS. Systemic ischemia/reperfusion leads to the activation of coagulation by the induction of TF on monocytes and endothelial cells, ultimately resulting in thrombin burst. DAMPs, including cfDNA, histones, and HMGB1, play a crucial role in the generation of thrombin *via* both TF-dependent pathways and XII-dependent pathways. The binding of thrombin to PARs produces several cytokines, which subsequently upregulate the expression of TF on endothelial cells and monocytes. Decreased levels of protein C and AT in circulation and reductions in AT, TM, TFPI, and EPCR on endothelial cells, which are caused by downregulation due to hypoxia and inflammatory cytokines and cleavage from the endothelium, are involved in the impairment of anticoagulant system. NE and the DAMP-mediated inhibition of the anticoagulant pathway also lead to the deterioration of the anticoagulant activity. t-PA is released from endothelial cells in the early phase of PCAS. PAI-1 increases 24 h after the onset of PCAS and keeps increasing in the late phase of PCAS, resulting in “no-reflow,” multiple organ dysfunction, and poor outcome. High concentrations of cfDNA also reduce the rate of fibrinolysis by competing for plasmin with fibrin. TM, thrombomodulin; PC, protein C; APC, activated protein C; EPCR, endothelial protein C receptor; AT, antithrombin; TFPI, tissue factor pathway inhibitor; PARs, protease-activated receptors; TF, tissue factor; HMGB1, high-mobility group box 1 protein; cfDNA, cell-free DNA; NE, neutrophil elastase; NETs, neutrophil extracellular traps; HMWK, high-molecular-weight kininogen; PAI-1, plasminogen activator inhibitor-1; t-PA, tissue-type plasminogen activator; Va, activated factor V; VIIa, activated factor VII; VIIIa, activated factor VIII; IX, factor IX; IXa, activated factor IX; X, factor X; Xa, activated factor X; XI, factor XI; XIa, activated factor XI; XII, factor XII; XIIa, activated factor XII; PCAS, post-cardiac arrest syndrome; DAMP, damage-associated molecular pattern. This figure was created by author.

**Figure 2:**
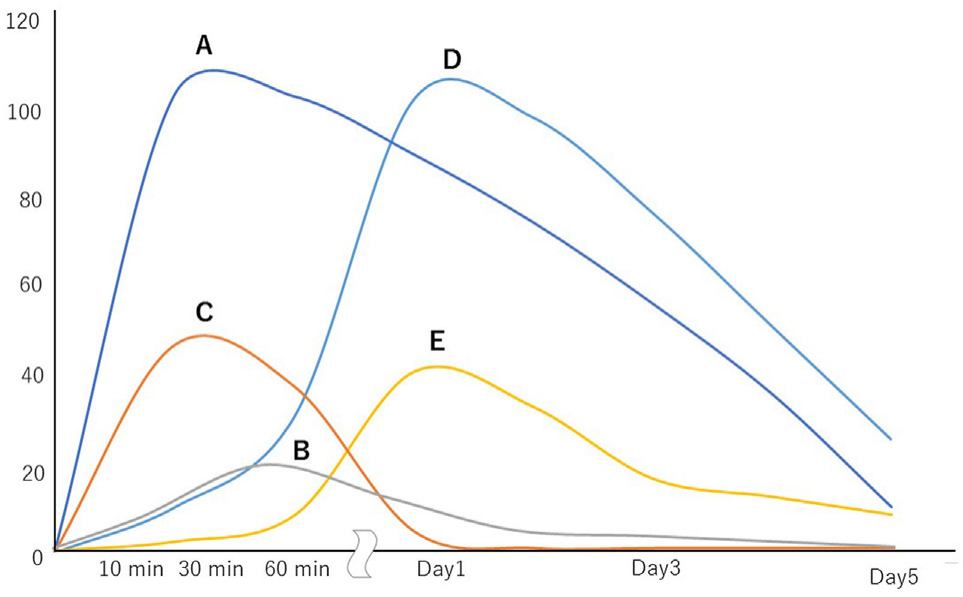
Chronological changes in the coagulofibrinolytic status of patients with post-cardiac arrest syndrome. The vertical axis shows the increases from the values of control subjects (times). A, thrombin activity; B, plasmin activity; C, tissue-type plasminogen activator activity; D, plasminogen activator inhibitor-1 activity; E, neutrophil elastase-mediated fibrinolytic activity.

A schematic illustration and the chronological changes in the coagulofibrinolytic status in patients with PCAS are shown in Figures 1 and 2, respectively.

### Coagulation System

#### Procoagulants in the Systemic Circulation

##### Tissue Factor-Initiated Coagulation

Patients with PCAS are in a condition of hypercoagulation, as has been confirmed by previous studies showing increased levels of soluble fibrin (11, 15), fibrinopeptide A (16), tissue factor antigen (17), and thrombin–antithrombin complex (12). Ischemia (hypoxia)/reperfusion due to cardiac arrest and ROSC and subsequent excessive catecholamine release due to shock-induced sympathoadrenal activation causes endothelial activation and injury. These changes induce the expression of tissue factor on the endothelial cells (18). Tissue factor exposed in the circulating blood binds to activated factor VII (FVIIa) (formerly known as the “extrinsic coagulation pathway”). This pathway is also facilitated by the exposure of perivascular tissue factor to the plasma compartment due to increased vascular permeability after ischemia/reperfusion (19). Tissue factor/FVIIa complex and ischemia itself activate factor X (FX), and the activated factor Va (FVa)/activated factor X (FXa) complex (FVa/FXa: prothrombinase) converts prothrombin into thrombin, which aggregates and activates platelets (20). Factors V, VIII, and IX in circulation bind to the activated platelets, resulting in a massive amount of thrombin generation (thrombin burst) by prothrombinase and activated factor VIII (FVIIIa)/activated factor IX (FIXa) complex (FVIIIa/FIXa: tenase) on activated platelets (20).

##### Inflammatory Cytokines

Inflammatory cytokines induce the expression of tissue factor in endothelial cells and monocytes (21). High levels of tumor necrosis factor-α (3, 22), interleukin (IL)-6 (3, 11), and IL-8 (3, 23) have been found in PCAS patients. Thrombin produced in this process not only converts fibrinogen to fibrin but also works as a potent pro-inflammatory factor through binding to protease-activated receptors (24), suggesting that systemic inflammation and coagulation activation shore up each other (11). On the basis of the concept that the pathophysiology in patients who achieve ROSC after cardiac arrest is similar to that observed in sepsis patients, Adrie et al. named this unique syndrome “sepsis-like syndrome” (3). Both patients with sepsis and patients with PCAS exhibited the high levels of cytokines in comparison to the healthy control subjects, suggesting that patients in both groups suffered from systemic inflammation due to severe insult; however, the levels of cytokines in the PCAS patients were lower than those in the sepsis patients (3). This can be explained by the difference in the degree and period of insult between sepsis and PCAS. Whether PCAS should be referred to as sepsis-like syndrome remains controversial.

##### Damage-Associated Molecular Patterns

Recent observations have highlighted the important role of DAMPs, which can be actively released by ischemic or impaired cells and be involved in the development of DIC (25, 26). PCAS patients were found to have high levels of DAMPs, including cell-free DNA (cfDNA) (27–30), DNA-binding proteins such as histones (DNA–histones complexes) (30), and high-mobility group box 1 protein (HMGB1) (31). cfDNA binds factor XII (FXII) and high-molecular-weight kininogen and triggers FXII- and factor XI-dependent blood coagulation (32–34).

A recent study showed a positive correlation to exist between thrombin generation and endogenous cfDNA and that histones and DNA–histone complexes also enhance thrombin generation in sepsis patients (34). Histones are a key component of neutrophil extracellular traps (NETs), which contribute to vascular thrombosis and inflammation (35). A study including both experimental and clinical aspects demonstrated that circulating histones directly induced features of thrombosis and DIC (36). Histones are also involved in platelet aggregation (37) and the release of pro-inflammatory cytokines (38). Furthermore, histone–DNA complexes might be important clinical prognostic markers and predictors of multiple organ failure and mortality in patients with DIC (39). HMGB1, which drives inflammation and tissue repair during its release into the extracellular milieu, is known as a mediator of lethal systemic inflammation in patients with sepsis (40, 41). Monocytes activated by extracellular HMGB1 show the expression of tissue factor on their surface (42). In addition, neutrophils activated by extracellular HMGB1 induce the extrusion of NETs (43).

Most of these studies on the relationship between DAMPs and coagulation were performed to investigate the pathophysiology of sepsis-associated coagulopathy. However, these findings can be applied to PCAS-related coagulopathy because PCAS patients have high levels of DAMPs.

#### Impairment of Endogenous Anticoagulant Activity

##### Endothelial Activation and Injury

The impairment of the physiological anticoagulant pathways results in a deterioration of the hypercoagulant state in patients with PCAS. Previous studies showed that PCAS patients have a high level of soluble P-selectin due to the activation of platelets, endothelial cells, and leukocyte activation, which is evidenced by a low level of soluble L-selectin (44, 45). In addition, PCAS patients were found to have high levels of soluble intercellular adhesion molecule-1, soluble vascular cell adhesion molecule-1, and E-selectin, which are the main adhesion molecules responsible for neutrophil attachment to endothelial cells (45, 46). These findings indicate that the pathophysiology of PCAS is involved in the neutrophil–endothelial interrelation followed by their activation, resulting in endothelial injury, which was confirmed by an increased level of soluble thrombomodulin (11, 44–48). These changes contribute to shedding, degradation, and/or the release of anticoagulants by endothelial cells, including thrombomodulin, endothelial protein C receptor, antithrombin, and TFPI (11, 17, 48, 49).

##### Protein C and Thrombomodulin

Protein C and its cofactor protein S constitute a defense line against the excessive activation of coagulation. Protein C is converted to its active form, activated protein C (APC) by thrombin–thrombomodulin complex on the endothelial cell surface. APC degrades FVIIIa and FVa, attenuating thrombin production and fibrin formation. Decreases in protein C antigen (50), protein C activity (11, 50), and protein S activity (11) were confirmed in successfully resuscitated patients. The decrease in protein C can be explained by massive thrombin formation following ROSC and consequent rapid consumption of protein C in the circulation (50). It should also be noted that DAMPs, such as histones and HMGB1, inhibit the thrombin–thrombomodulin complex-mediated anticoagulant protein C pathway (42, 51).

##### Antithrombin

Antithrombin, which forms complexes with thrombin and inhibits thrombin and FXa, is one of the important anticoagulant factors. Previous studies have confirmed reduced levels of antithrombin in PCAS patients (11, 52), particularly those with DIC (53), with a poor outcome (54), and with refractory shock (11). The reduction in the antithrombin levels may be caused by not only consumption through the formation of complexes with thrombin and protease but also extravascular loss due to increased vascular permeability (20, 55). In addition, degradation of antithrombin by neutrophil elastase (NE) may be a cause of antithrombin reduction (56).

##### Tissue Factor Pathway Inhibitor

Tissue factor pathway inhibitor is a major inhibitor of tissue factor-initiated coagulation. This inhibitor is bound to the endothelial surface and lipoproteins in the circulation. Low TFPI levels have been found in PCAS patients (17). High levels of NE, which cleaves TFPI, were confirmed in patients with PCAS in previous studies (15, 22, 46, 57). Therefore, NE may be one reason why the TFPI levels are decreased in patients with PCAS, which impairs the ability of TFPI to counteract tissue factor activity.

As a result of these changes, the anticoagulant system is impaired, leading to the acceleration of thrombin generation.

### Fibrinolytic System

#### Acute Release of t-PA

In the early phase of PCAS, hyperfibrinolysis was confirmed by marked increases in t-PA antigen and activity (16) and high levels of plasmin-alpha2 plasmin inhibitor complex (a marker of plasmin formation) (11, 15, 52), d-dimer (11, 15, 52), and fibrin/fibrinogen degradation product (FDP) (15, 54), as well as based on the results of rotational thromboelastometry (ROTEM) (58). Whole-body ischemia/reperfusion and tissue hypoxia due to ROSC after cardiac arrest cause t-PA release from Weibel–Palade bodies in endothelial cells, leading to systemic hyperfibrinolysis (59, 60). A recent study demonstrated that the lactate levels, which reflect the degree of tissue hypoperfusion, is an independent predictor of developing PCAS-related DIC with hyperfibrinolysis (53). This result is also supported by other studies regarding the significant relationship between hypoperfusion markers—including pH, base excess, and lactate—and hyperfibrinolysis detected by ROTEM in PCAS patients (61) and hyperfibrinolytic DIC resulting from asphyxia by drowning (62). Interestingly, no t-PA release was detected in the late phase of PCAS, leading to MODS caused by an imbalance between the activation of coagulation and the activation of endogenous fibrinolysis (12, 16).

#### Fibrinolytic Shutdown

Plasminogen activator inhibitor-1 is the primary inhibitor of t-PA. Fibrinolytic shutdown due to increases in PAI-1 is a factor leading to the “no-reflow phenomenon” (12, 15). Contrary to t-PA, PAI-1 is not stored within endothelial cells but is instead found in plasma, platelets, and extracellular matrix (63). However, PAI-1 mRNA is expressed under hypoxic conditions, and PAI-1 antigen appears within 6 h after hypoxia, with the peak levels achieved at 20–24 h after hypoxia (11, 63, 64). This increase in PAI-1, which has been confirmed at 24 h after the onset of PCAS (late phase of PCAS, not early phase), leads to fibrinolytic shutdown, resulting in MODS and a poor outcome (15, 65). In the early stage of PCAS, a moderate increase in the PAI-1 antigen has also been found (16). This increase may be due to the thrombin-activated release from platelets.

Interestingly, DAMPs appear to be involved in the development of fibrinolytic shutdown. Physiologically, cfDNA enhances the activation of plasminogen by t-PA and concurrently suppresses fibrinolysis by potentiating the inactivation of t-PA by PAI-1 (66). However, higher concentrations of DNA attenuate fibrinolysis (66). In addition, DNA-histone complexes and histones make fibrin fibers thicker, resulting in the formation of more stable clots that are resistant to shear force (67).

#### Neutrophil-Mediated Fibrinolysis

Insufficient activation of fibrinolysis may also be associated with the pathophysiology of organ dysfunction in PCAS (15). Not only plasmin but also NE mediates the degradation of fibrin(ogen). The products degraded by NE (fibrin degradation product by NE, EXDP) are distinguished from plasmin digest (68, 69). A previous study revealed significant correlations between the levels of PAI-1 and EXDP in PCAS patients without MODS, while there were no significant correlations between these values in patients with MODS. This result suggests that PCAS patients may be able to avoid developing MODS if NE-mediated fibrinolysis can make up for the fibrinolytic shutdown by PAI-1. However, the levels of NE in PCAS patients with MODS were high in comparison to those without MODS (15). These conflicting results may be explained by the noted evidence, which suggested that the changes in the levels of NE and EXDP differed from those observed in sepsis-induced DIC (69). The precise role of NE in the pathophysiology of PCAS-associated DIC remains unclear.

### Differences in Coagulofibrinolytic Changes by Causes of Cardiac Arrest

**Figure 3:**
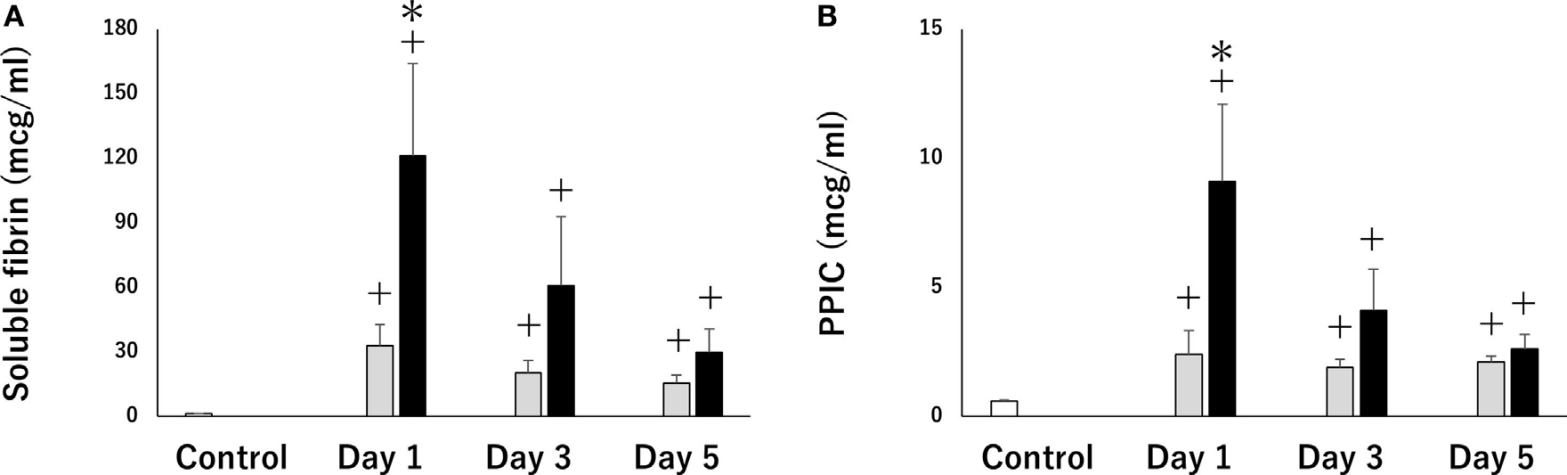
Serial changes in soluble fibrin **(A)** (a marker of thrombin activation) and plasmin-α2 plasmin inhibitor complex (PPIC) **(B)** (a marker of plasmin activation). Thirteen patients with post-cardiac arrest syndrome (PCAS) caused by cardiogenic cardiac arrest (black bars) and 13 patients with PCAS caused by hypoxia-related cardiac arrest were enrolled. The white bars represent control subjects (healthy adult). All results were expressed as the mean ± SEM. ^+^*p* < 0.05 vs control subjects, *p < 0.05 cardiogenic group vs hypoxia group.

A recent study indicated that PCAS patients who suffer from cardiac arrest due to a hypoxic event exhibited severe thrombin activation and hyperfibrinolysis in comparison to patients who experience cardiac arrest due to a cardiogenic cause (Figure 3) (70). This result coincides with a previous report showing that asphyxia by drowning leads to severe hemorrhage due to hyperfibrinolytic DIC (62). Hypoxic PCAS is influenced by both hypoxia caused by circulatory arrest and pre-cardiac arrest hypoxia, followed by more serious endothelial damage and coagulopathy. These results may be directly supported by the results of previous studies, indicating that the time from the onset of cardiac arrest to first CPR and the duration of CPR were primary causes of hyperfibrinolysis (61, 62, 71, 72) and that poor cerebral oxygenation during CPR in out-of-hospital cardiac arrest (OHCA) are associated with hyperfibrinolysis (72), suggesting that the degree of hypoxia is a significant determinant of the severity of PCAS-related DIC.

### The Changes in the Coagulation System Due to Targeted Temperature Management (TTM)

Targeted temperature management is one of major standard therapeutic options for PCAS because it can improve survival and the neurological outcomes in PCAS patients through the attenuation of neurological injury caused by hypoxia and reperfusion injury (2, 73). However, as previous studies on the relationship between hypothermia and poor outcomes in patients with sepsis and trauma have shown (74, 75), TTM for PCAS may be associated with impairment of hemostasis, which may be followed by uncontrolled bleeding, especially in PCAS patients who have suffered thromboembolic cardiac events because they require effective antiplatelet therapy after primary percutaneous coronary intervention (76).

Nielsen et al. investigated the changes in the coagulation of PCAS patients during TTM using ROTEM and standard coagulation tests. They found that coagulation in hypothermia (33°C) was not substantially different from coagulation in normothermia (37°C) (77). Another previous study by Jacob et al. investigated whether the application of different levels of TTM (33 and 36°C) to patients with PCAS due to presumed myocardial infarction affected the coagulation system with regard to standard laboratory coagulation parameters and thromboelastography. The study found no evidence to suggest that 33°C temperature management has an adverse effect on coagulation or bleeding (76). These findings indicate that TTM does not impair coagulation and that TTM can be safely applied to PCAS patients including those who require antithrombotic therapy.

## Predicting the Outcome of PCAS Patients

Numerous studies have investigated the factors associated with the risk of death in PCAS patients. Previous reports investigating the relationship between the risk of death in PCAS patients and the levels of d-dimer showed that the levels of d-dimer on admission were independent predictors of the 30-day mortality in patients with OHCA (78) and that the precardiac arrest d-dimer level could be a valuable predictor of immediate mortality after ROSC in patients with in-hospital cardiac arrest (79). A recent study investigated the correlation between the coagulofibrinolytic values and the outcome of PCAS patients combined with prehospital information. In this study, there were significant positive correlations between time from calling emergency medical services to ROSC and the FDP levels on admission, for which the cutoff point was able to predict a favorable neurological outcome with high accuracy (54).

DIC scores were also proven to be useful predictive factors for the outcome of PCAS patients. The Japanese Association for Acute Medicine DIC diagnostic criteria (80) independently predicted the 28-day mortality (53) and the severity of organ dysfunction associated with PCAS (15), which was evaluated in accordance to the Sequential Organ Failure Assessment scores (81). A high initial International Society of Thrombosis and Haemostasis DIC score was also an independent risk factor of both in-hospital mortality and an unfavorable outcome (71).

## Coagulofibrynolitic System as a Therapeutic Target for PCAS

Representative experimental studies on coagulofibrinolytic changes associated with cardiac arrest and the “no-reflow phenomenon” and prospective clinical trials on thrombolytic therapy in PCAS are summarized in Tables 1 and 2, respectively.

**Table 1:** Experimental studies on coagulofibrinolytic changes associated with cardiac arrest and “no-reflow phenomenon.”

Authors	Intervention	Method	Effect
Ames et al. (8)	Heparin	Cerebral ischemia in rabbits	Extended period of permissible ischemia and long-term survival
Kowada et al. (82)			
Fischer et al. (83)	Hemodilution with saline	Cerebral ischemia in rabbits	Improvement of cerebral circulatory impairment
Safar et al. (84)	Combination of hypertensive perfusion, heparin, and dextran	Cardiac arrest in dogs	Improvement of neurological deficit score and normalization of the electroencephalogram
Lin et al. (85)	Combination of dextran and streptokinase	Cardiac arrest in dogs	Improvement of impaired cerebral blood flow and normalization of the electroencephalogram
Fischer et al. (88)	Heparin and recombinant tissue plasminogen activator	Cardiac arrest in cats	Improvement of microcirculatory reperfusion
Teschendorf et al. (109)	Recombinant human activated protein C	Cardiac arrest in rats	No deference of neurological deficit score. Failure to limit the inflammatory response to ischemic injury
Teschendorf et al. (110)	Recombinant human activated protein C	Cardiac arrest in rats	No significant effect on wall shear rate and plasma extravasation
Johansson et al. (111)	Antithrombin	Cardiac arrest in piglets	Failure to increase cerebral circulation or reduce reperfusion injury
Yin et al. (114)	Shen-Fu injection (ginsenoside and acotinine)	Cardiac arrest in pigs	Inhibition of coagulation–fibrinolysis disorders after cardiac arrest

**Table 2:** Representative prospective clinical trials on thrombolytic therapy in post-cardiac arrest syndrome.

Authors	Design	Subject	Thrombolytic drug	Outcome	Hemorrhagic complication
Böttiger et al. (91)	Prospective observational trial	OHCA	t-PA	ROSC: 68 vs 44% (*p* = 0.026)	2 patients with thrombolytic required transfusion vs 0 with control (*p* = 0.379)
				Admission to cardiac ICU: 58 vs 30% (*p* = 0.009)	
				Survival up to 24 h: 35 vs 22% (*p* = 0.171)	
				Survival till discharge: 15 vs 8% (no *p* value provided)	
				All above are thrombolytic group vs control group	

Abu-Laban et al. (92)	Prospective, randomized, placebo-controlled trial	PEA unresponsive to initial therapy	t-PA	No significant difference in outcome, including ROSC, length of hospital stay, and survival to hospital discharge	2 major hemorrhage with thrombolytic vs 1 major and 1 minor hemorrhage with control (*p* = 0.50 and *p* = 0.99, respectively)

Böttiger et al. (97)	Prospective, randomized, placebo-controlled trial	Witnessed OHCA due to presumed cardiac causes	Tenecteplase	No significant differences in 30-day survival, hospital admission, ROSC, 24-h survival, survival to hospital discharge, or neurologic outcome	Intracranial hemorrhage: 2.7% with thrombolytics vs 0.4% with control (*p* = 0.006)

*OHCA, out-of-hospital cardiac arrest; PEA, pulseless electrical activity; t-PA, tissue-type plasminogen activator; ROSC, return of spontaneous circulation*.

### Experimental Studies on Therapeutic Targets for the “No-Reflow Phenomenon”

At the same time as the concept of “no-reflow phenomenon” was born, researchers naturally tried to develop therapy to combat this novel disease. Ames et al., who reported the “no-reflow phenomenon” for the first time, suggested that heparin might help restore the blood pressure after ischemia/reperfusion to obtain a slightly better recovery and long-term survival (8, 82). They also exhibited the importance of hemodilution with saline, based on their experimental finding that the major cause of post-ischemic vascular impairment was erythrocyte aggregation, which is followed by increased blood viscosity in no-flow conditions (83). Thereafter, improved cerebral outcome after cardiac arrest was observed using a combination of hypertensive reperfusion, heparin, and dextran (84). Dextran was also found to improve the post-cardiac arrest cerebral blood flow when administered in combination with streptokinase (85).

### Thrombolytic Therapy

Based on the results of experimental and clinical studies showing the efficacy of the administration of thrombolytic agents, such as t-PA, urokinase, and streptokinase, in the pathology of PCAS (12, 86–90), the first prospective study of t-PA in cardiac arrest was conducted. This study demonstrated that thrombolytic therapy (t-PA combined with heparin in this trial) had a strong association with an improvement of mortality along with a favorable safety profile (91). However, the first prospective randomized, blinded, placebo-controlled trial in OHCA patients whose initial rhythm was pulseless electrical activity showed no evidence of a favorable effect of t-PA (92). Although some researches have reported the availability and safety of thrombolytic therapy in OHCA patients (93–96), a randomized, blinded, placebo-controlled trial assessing patients with witnessed OHCA due to presumed cardiac causes demonstrated no marked difference in the survival outcomes between tenecteplase and placebo. Furthermore, intracranial hemorrhaging occurred with significantly higher frequency in the tenecteplase group than in the placebo group. One possible reason for the apparent ineffectiveness of tenecteplase was suggested to be the fact that antithrombotic and antiplatelet agents were not given during CPR or before arrival at the hospital due to concern about the bleeding risk (97). Since then, several studies encouraging the use of t-PA have been published (98, 99), but no guidelines or studies with high evidence levels supporting thrombolytic therapy in PCAS have yet been published. The guidelines for CPR and emergency cardiovascular care, which were published by the American Heart Association in 2015, recommended thrombolytic therapy as a reasonable emergency treatment option for patients with confirmed pulmonary embolism as the precipitant of cardiac arrest (Class IIa). These guidelines do not mention thrombolytic therapy as a management in PCAS patients in whom the cause of cardiac arrest is undetermined (100).

### Anticoagulant Agents

A lot of experimental studies showing the efficacy of anticoagulants in treating critical organs with ischemia/reperfusion injury have been reported. APC was proven to attenuate ischemia/liver (101), spinal cord (102), and brain (103, 104). Antithrombin also attenuated ischemia/reperfusion injury in the intestines (105) and kidneys (106). In addition, recent studies have suggested that both antithrombin and APC are protective against myocardial ischemia and reperfusion injury (107, 108). To our best knowledge, however, no studies have been shown the beneficial effects of these anticoagulant properties on systemic ischemia/reperfusion injury. Quite to the contrary, the findings from two experimental studies in rats do not support the effect of protein C on PCAS pathology (109, 110), and the administration of antithrombin offered no positive effects on the cerebral circulation and reperfusion injury in piglets (111). Recent studies have indicated that these anticoagulants exert not only antithrombotic effects but also anti-inflammatory effects. In particular, there have been some clinical investigations showing the efficacy of antithrombin in the treatment of DIC associated with sepsis (112, 113). One possible explanation for the failure of previous studies to prove the effectiveness of these agents against PCAS pathology may be pathophysiologic differences between sepsis and PCAS (110). Ischemic injury and the subsequent inflammatory and coagulation reactions may not be of sufficient severity for anticoagulant agents to show a beneficial effect (109). Other possible reasons may include the limited intensity of inflammation and coagulation in the model used in these studies and the dose, timing, or/and duration of drug administration (109, 111). A clinical study should be performed to clarify the effects of anticoagulant agents in PCAS patients.

### Others

Urinastatin, a trypsin inhibitor, suppresses the increase in elastase by direct proteolysis, neutrophil inhibition, and suppression of elastase release from activated neutrophils. A prospective single-center randomized trial showed that while the administration of urinastatin significantly inhibited the increase in elastase after ROSC, it failed to improve the clinical outcome (57).

Recent studies on Shen-Fu injection have produced interesting findings. This is the typical form of Shen-Fu decoction, which has been used in China for a long time. The main active ingredients of Shen-Fu injection are ginsenoside and aconitine (114). Previous studies have reported that Shen-Fu injection can minimize PCAS-associated brain edema (115), myocardial dysfunction (116), and lung injury (117). Shen-Fu injection was also found to inhibit the coagulofibrinolytic changes associated with PCAS, which might be involved in attenuating endothelial dysfunction and improving the systemic metabolism (114). Clinical trials should be performed to clarify the pathological effectiveness of Shen-Fu injection in patients with PCAS because all of the above results have been based on animal experiments.

## Conclusion

Coagulofibrinolytic changes in patients with PCAS are characterized by the hypercoagulative state, which is accelerated by impaired anticoagulant activities, and hyperfibrinolysis in the super-early phase of PCAS, followed by inadequate endogenous fibrinolysis and fibrinolytic shutdown by PAI-1. These changes are deteriorated by inflammatory cytokines released from monocytes and DAMPs released from damaged cells. Substantial evidence supporting this unique pathophysiology has been accumulated since the “no-reflow phenomenon” was first reported around half a century ago, but the optimum therapeutic strategy has yet to be established. Unfortunately, research in this field appears to be declining, although the academic area of coagulation and fibrinolysis has been developing over the past decade. This review will hopefully encourage more research into this subject.

## Author Contributions

The author searched the literature, reviewed the papers, and drafted the manuscript.

## Conflict of Interest Statement

The author declares that the research was conducted in the absence of any commercial or financial relationships that could be construed as a potential conflict of interest.
